# Sex (as recorded) of anesthesia providers and perioperative outcomes: a systematic review and meta-analysis

**DOI:** 10.1016/j.bjane.2026.844745

**Published:** 2026-03-06

**Authors:** Tauãna T.C. de Oliveira, Vanessa Tapioca, Sara Amaral, Bruna Ferreira, Fernanda Marinho, Sarah Saxena

**Affiliations:** a Universidade Federal de São João del-Rei, Department of Medicine, São João del-Rei, MG, Brazil; b Duke University Medical Center, Department of Anesthesiology, North Carolina, USA; c Universidade da Região de Joinville, Department of Medicine, Joinville, SC, Brazil; d Faculdade de Ciências da Saúde Dr. José Antônio Garcia Coutinho, Department of Medicine, Pouso Alegre, MG, Brazil; e University of Mons, Research Institute for Health Sciences and Technology, UMons, Department of Surgery, Mons, Belgium; f Helora, Department of Anesthesiology, Mons, Belgium

Dear Editor,

There has been increasing interest in whether clinician sex is associated with patient outcomes in perioperative care. Evidence from surgical fields suggests that provider-level factors, including surgeon sex, may be associated with postoperative outcomes.^1^ In contrast, anesthesiology differs from surgery in several structural and clinical aspects that may limit provider-related variability in outcomes. Anesthesia care is commonly delivered within supervised, team-based models and guided by standardized monitoring and protocol-driven practice, which may reduce individual provider-related variability in perioperative outcomes compared with surgical practice.^2^^,^^3^ Nevertheless, whether anesthesia provider sex is associated with measurable differences in perioperative outcomes remains unclear.

To address this gap, we performed a systematic search of PubMed, Embase, and Scopus from inception to November 2025. A detailed search strategy is available in Table S1. We included studies enrolling adult (≥ 18 years) surgical patients who received any type of anesthesia delivered by female or male anesthesiologists, anesthesiology residents or fellows, or certified registered nurse anesthetists. The review followed the Preferred Reporting Items for Systematic Reviews and Meta-Analyses (PRISMA) guidelines.^4^ (PROSPERO: CRD420251166322). We excluded studies with overlapping populations from the same study centers or cities, retaining the study with the largest population when overlap was identified, and studies that did not compare anesthesia provider sex.

We selected all-cause 30-day mortality as the main outcome because it is a clinically meaningful endpoint commonly reported in perioperative cohorts. Intraoperative hypotension and postoperative renal complications were chosen as additional outcomes because they are commonly reported perioperative quality indicators and were available across the included cohorts.

Effect estimates were extracted from adjusted models and aligned to a common comparator (male vs female anesthesia provider). For the purpose of quantitative synthesis, we attempted to harmonize outcome definitions across studies. Hypotension was considered present when reported as either an episode requiring vasopressor treatment or a mean arterial pressure < 65 mmHg. Postoperative renal complications were defined as any reported impairment in renal function, and mortality as all-cause death within 30 days after surgery. When multiple definitions were available, the outcome most closely matching this definition was extracted. Detailed outcome definitions for each study were provided in Table S2.

Adjusted effect estimates were extracted from multivariable models in each study. The covariate sets differed across studies, typically including combinations of patient characteristics, procedural variables, and provider-related factors. These differences in model specification may affect the comparability of adjusted estimates and should be considered when interpreting pooled results. Detailed adjustment variables for each study are provided in Table S3.

Two authors (TT and BF) independently screened studies and extracted data, while two others (VT and FM) independently assessed risk of bias using ROBINS-I tool (Fig. S1).^5^ Statistical analyses were conducted using Review Manager 5.4.1 software (Nordic Cochrane Centre, The Cochrane Collaboration, 2014; Copenhagen, Denmark). Adjusted estimates were log transformed with corresponding standard errors and pooled as Odds Ratios (OR) with 95% Confidence Intervals using a random-effects model (p < 0.05).

We included four studies,^6^, ^7^, ^8^, ^9^, comprising 1,632,086 patients in total, of whom 537,681 (32.9%) received care from female anesthesia providers. Table 1 and Figure S2 present the key characteristics of the included studies and the corresponding study selection process, respectively. Across included studies, anesthesia provider sex was recorded as a binary variable (female vs. male), obtained either from administrative databases or self-reported at credentialing or employment. Gender identity was not collected in the included datasets. These differences in data sources should be considered when interpreting sex-based comparisons across studies.

**Table 1:** Baseline characteristics of the included studies.

Author, year	Country	Female / Male providers (%)	Patients cared by a female / male provider (n)	ASA status	Type of anesthesia provider	Anesthesia provider sex	Type of surgery	Duration of surgery [min] Mean ± SD
Chui, 2025	Canada	NA	1,275 / 6,417	I = 131	Attending anesthesiologist; Trainee (resident, fellow); Neuroanesthesia fellowship-trained anesthesiologist; Canadian-certified anesthesiologist	Sex was recorded as a binary variable and was extracted from the institutional administrative database, although authors use the term gender in the supplementary material.	Instrumentation and fusion; Lumbar discectomy and/or laminectomy; Spinal tumor; Kyphosis or scoliosis correction	145.0 ± 90.0
				II = 1,377				
				III = 4,563				
				IV = 1,593				
				ND = 20				
Jerath, 2024	Canada	31.5% / 68.5%	311,822 / 853,889	NA	Attending anesthesiologist (specialty-trained physician); Trainee (resident, fellow)	Anesthesia provider sex was self-reported at the time of credentialing and obtained from the Corporate Provider Database. Sex was recorded as a binary variable (female vs. male), while provider gender was not collected.	Cardiovascular; General Surgery; Neurosurgery; Obstetrics and gynecology; Otolaryngology; Orthopedic; Plastic; Thoracic; Urology; Vascular	Female 124.9 ± 99.6
								Male 122.4 ± 105.8
von Wedel, 2024	USA	43.2% / 56.8%	185,170 / 179,259	I = 46,135	Trainee (resident, fellow); CRNA; Attending anesthesiologist	Anesthesia provider sex was self-reported upon employment and obtained from the employee database. Sex was recorded as a binary variable (female vs. male), while the provider’s gender was not collected.	Cardiac surgery; Cardiology; Colorectal; Ear, nose, throat Gastrointestinal; General surgery; Gynecology and obstetrics Neurosurgery; Ophthalmology; Orthopedic surgery; Plastic surgery; Podiatry; Surgical oncology; Thoracic; Trauma; Transplant; Urology; Vascular	Female 113.7 ± 85.2
				II = 179,632				Male 126.6 ± 80.8
				III = 117,887				
				IV = 20,775				
Zeiner, 2024	Austria	41.8% / 58.2%	39,414 / 54,840	I = 24,732	Anesthesiologists (physicians)	Anesthesia provider sex was self-reported biological sex, extracted from institutional provider databases. Sex was recorded as a binary variable (female vs. male), and provider gender was not collected.	Cardiothoracic; Dermatological; Ear, nose and throat; General surgery; Gynecology; Maxillofacial; Neurosurgery; Non-operating-room anesthesia; Obstetric; Orthopedic; Robotic; Trauma; Urology; Vascular	Female 138.0 ± 102.0
				II = 36,779				Male 144.0 ± 102.0
				III = 27,676				
				IV = 4,338				
				V = 725				

ASA, American Society of Anesthesiologists; CRNA, Certified Registered Nurse Anesthetist; NA, Not Available; ND, Not Documented/unclear.

Anesthesia provider sex was not associated with statistically significant differences in all-cause 30-day mortality (OR = 1.04 [95% CI 0.97–1.11]; p = 0.32, *I²* = 84%, n = 1,624,394; Fig. 1A), intraoperative hypotension (OR = 1.02 [95% CI 1.00–1.04]; p = 0.10, *I²* = 52%, n = 458,683; Fig. 1B), or postoperative renal complications (OR=1.00 [95% CI 0.98–1.02]; p = 0.98, *I²* = 0%, n = 101,946; Fig. 1C). Sample sizes differed across outcomes because not all studies contributed data to each synthesis. For all-cause 30-day mortality, Chui et al.^6^ was excluded due to likely population overlap with Jerath et al.,^7^ and the larger cohort (Jerath et al.) was retained. The overall risk of bias was moderate (Fig. S1), mainly due to residual confounding inherent to the observational design.

**Figure 1:**
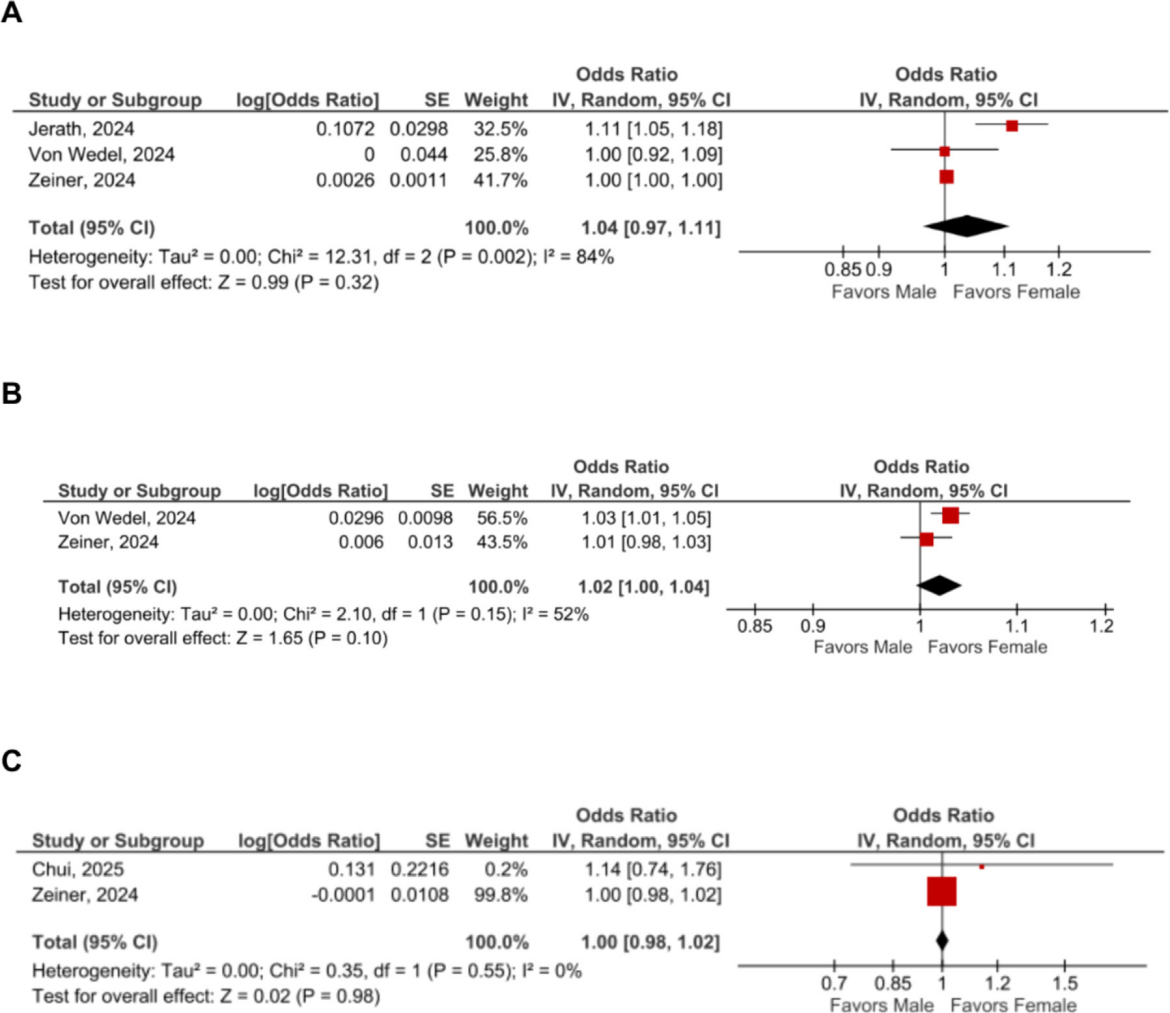
Forest plots of the effect of anesthesia provider sex on (A) All-cause 30-day mortality, (B) Intraoperative hypotension, and (C) Postoperative renal complications. The plot displays odds ratios with 95% Confidence Intervals for each included study and the pooled random-effects estimate. CI, Confidence Interval; SE, Standard Error.

Few studies have examined provider sex in anesthesia, and this meta-analysis did not demonstrate statistically significant differences in all-cause 30-day mortality, intraoperative hypotension, or postoperative renal complications based on anesthesia provider sex. To our knowledge, this is the first meta-analysis assessing whether anesthesia provider sex is associated with perioperative outcomes, a question of increasing relevance amid ongoing scrutiny of gender equity in perioperative care. Women remain underrepresented in perioperative roles and continue to face disparities in income, leadership opportunities, and career advancement.^10^ Understanding whether such structural inequities translate into differences in clinical practice or patient outcomes carries both scientific and policy relevance.

In contrast to evidence from surgery, where a recent meta-analysis reported lower postoperative mortality among patients treated by female surgeons^1^ we found no statistically significant differences in all-cause 30-day mortality, intraoperative hypotension, or postoperative renal complications based on anesthesia provider sex. Differences in surgical and anesthetic practice structures may partly explain these discrepant findings. The team-based organization of anesthesia care may attenuate individual provider effects on outcomes, as prior evidence shows similar perioperative outcomes across anesthesia care team models.^3^

Despite the high degree of standardization of anesthetic protocols, substantial heterogeneity was observed across most analyses, particularly for mortality (Fig. 1A) and intraoperative hypotension (Fig. 1B), limiting the interpretability of a single pooled estimate and highlighting the need to move beyond average effects. This heterogeneity should also be interpreted considering differences in covariate adjustment across studies. Because only four studies were included, subgroup analyses or meta-regression were not performed. As these outcomes are associated with multiple patient-, procedure-, and system-level factors, a precise pooled average may obscure clinically relevant, context-specific variations related to case-mix, institutional practices, and residual confounding. Additionally, international differences in the organization of anesthesia care, such as varying levels of autonomy among trainees, could have contributed to the heterogeneity observed across studies. In contrast, no statistical heterogeneity was observed for postoperative renal complications (Fig. 1C), which may reflect more consistent outcome definitions and protocol-driven kidney protection strategies; however, this finding should be interpreted cautiously given the limited number of contributing studies.

Although the included studies classified providers by sex, gender inequities represent broader structural and social dynamics, which are not captured by binary sex variables. The absence of measurable differences in clinical outcomes does not indicate that gender inequities in anesthesiology are inconsequential. Persistent gaps in representation, remuneration, and career progression remain well documented.^10^ Our findings suggest these disparities are unlikely to reflect differences in clinical performance, highlighting the need to address structural, cultural, and institutional factors within the specialty.

Our analysis offers an important first step toward understanding the interface between provider sex dynamics and perioperative care quality. As interest in the association between clinician sex and patient outcomes continues to grow, better-designed, adequately powered studies will be essential to refine these estimates and explore potential sex- or gender-related differences in more nuanced domains of anesthetic care.

This meta-analysis has several limitations. First, the evidence base comprised only four observational studies, limiting the robustness of pooled estimates and increasing the risk of publication bias. In the absence of randomized trials, residual confounding remains likely despite adjustment for patient-, provider-, and procedure-level variables. Second, we assessed a binary sex variable that does not capture the complexity of gender identity or gendered experiences, which reflect broader structural and social dynamics and may be associated with differences in communication, decision-making, and team interactions. Third, substantial heterogeneity across studies may reflect differences in healthcare systems, provider qualifications, and perioperative workflows, limiting generalizability. Finally, variations in outcome definitions and measurement methods may have contributed to between-study variability in pooled estimates.

Our findings suggest that within highly protocol-driven anesthesia practice, provider sex does not appear to be associated with clinically meaningful differences in short-term perioperative outcomes at the population level. Future studies with more comprehensive demographic data, broader clinical settings, and more nuanced outcomes will be essential to clarify whether sex and gender are associated with differences in the delivery of perioperative care.

## Data availability statement

The data that support the findings of this study are available from the corresponding author upon reasonable request.

All authors (1) Read and approved the final version, (2) Met the ICMJE criteria for authorship, (3) Believe the paper represents honest work, and (4) Are able to verify the validity of the results reported.

## Disclosures

SS is the lead of ESAIC’s subcommittee for the Geriatric Patient and has received speaker’s fees from Medtronic/Merck. All other authors declare no conflicts of interest. A preliminary version of this study was previously presented as an abstract at the ASA Annual Meeting 2025. The authors received no financial support for the submitted work.

## Authors' contributions

Conception and design of the study: TT, SA, SS.

Data acquisition and analysis: TT, VT, BF, FM.

Data interpretation: All authors.

Drafting the manuscript or revising it critically for important intellectual content: All authors.

Final approval of the version to be submitted: All authors.

Agreement to be accountable for all aspects of the work, thereby ensuring that questions related to the accuracy and integrity of any part of the work are appropriately investigated and resolved: All authors.

## Funding

This research did not receive any specific grant from funding agencies in the public, commercial, or not-for-profit sectors.

## Conflicts of interest

The authors declare no conflicts of interest.

## Appendix Supplementary materials

Image, application 1

## Footnotes

Supplementary material associated with this article can be found in the online version at doi:10.1016/j.bjane.2026.844745.
