# Supplementary material for: Sex (as recorded) of anesthesia providers and perioperative outcomes: a systematic review and meta-analysis
Source: Braz J Anesthesiol. 2026 Mar 6;76(3):844745. doi: 10.1016/j.bjane.2026.844745 (PMC13091334; doi:10.1016/j.bjane.2026.844745)
Supplement: Supplementary file 1 [file mmc1.docx]

**BJAN-D-26-00058 ‒ Supplementary Material**

**Supplementary Material (SM) For:**

**Sex (as recorded) of anesthesia providers and perioperative outcomes: a systematic review and meta-analysis**

1. **Table S1**. Complete search strategy.
2. **Table S2**. Outcome definitions for individual studies.
3. **Table S3.** Covariates included in adjusted models across included studies.
4. **Figure** **S1.** Risk of bias assessment using the ROBINS-I tool for non-randomized studies.
5. **Figure** **S2.** PRISMA flow diagram of study screening and selection.

**Table S1** Complete search strategy.

| **Embase search strategy:** | ('anesthesiology'/exp OR 'anesthesia'/exp OR 'anesthesiologist'/exp OR 'anaesthesiology' OR 'anaesthesia' OR 'anesthesiologist' OR 'anaesthesiologist' OR 'neuroanesthesia' OR 'anesthesiologist-surgeon dyad' OR 'anesthesiologist-surgeon collaboration' OR 'female anesthesiologist' OR 'female anaesthesiologist' OR 'women anesthesiologists' OR 'women anaesthesiologists' OR 'female anesthesiologist*' OR 'women anesthesiologist*' OR 'female physician' OR 'female surgeon' OR 'women physician*') AND ('provider sex' OR 'gender disparity' OR 'provider gender' OR 'dyad volume' OR 'provider role' OR 'gender differences' OR 'gender disparity in healthcare' OR 'gender differences in surgery' OR 'anesthesiologist sex') AND ('surgery'/exp OR 'operative procedures' OR 'surgery' OR 'perioperative complications' OR 'surgical complications' OR 'operative outcomes' OR 'surgical outcomes' OR 'surgical techniques' OR 'operative techniques' OR 'postoperative complications' OR 'population-based') |
| --- | --- |
| **Pubmed search strategy:** | ("Anesthesiology"[Mesh] OR "Anesthesia"[Mesh] OR "Anesthesiologists"[Mesh] OR Anaesthesiology OR anaesthesia OR Anesthesiologist OR Anaesthesiologist OR Neuroanesthesia OR "Anesthesiologist-Surgeon Dyad" OR "Anesthesiologist-Surgeon Collaboration" OR "female anesthesiologist" OR "female anaesthesiologist" OR "women anesthesiologists" OR "women anaesthesiologists" OR "female anesthesiologist*" OR "women anesthesiologist*" OR "female physician" OR "female surgeon" OR "women physician*”) AND (“provider sex" OR "gender disparity" OR "provider gender" OR "dyad volume" OR "provider role" OR "gender differences" OR "gender disparity in healthcare" OR "gender differences in surgery" OR “Anesthesiologist Sex”) AND ("Surgical Procedures, Operative"[Mesh] OR "operative procedures" OR "surgery" OR "perioperative complications" OR "surgical complications" OR "operative outcomes" OR "surgical outcomes" OR "surgical techniques" OR "operative techniques" OR "postoperative complications" OR "population-based") |
| **Scopus search strategy:** | TITLE-ABS-KEY ("Anesthesiology" OR "Anesthesia" OR "Anesthesiologists" OR Anaesthesiology OR anaesthesia OR Anesthesiologist OR Anaesthesiologist OR Neuroanesthesia OR "Anesthesiologist-Surgeon Dyad" OR "Anesthesiologist-Surgeon Collaboration" OR "female anesthesiologist" OR "female anaesthesiologist" OR "women anesthesiologists" OR "women anaesthesiologists" OR "female anesthesiologist*" OR "women anesthesiologist*" OR "female physician" OR "female surgeon" OR "women physician*") AND TITLE-ABS-KEY ("provider sex" OR "gender disparity" OR "provider gender" OR "dyad volume" OR "provider role" OR "gender differences" OR "gender disparity in healthcare" OR "gender differences in surgery" OR "Anesthesiologist Sex") AND  TITLE-ABS-KEY ("Surgical Procedures, Operative" OR "operative procedures" OR "surgery" OR "perioperative complications" OR "surgical complications" OR "operative outcomes" OR "surgical outcomes" OR "surgical techniques" OR "operative techniques" OR "postoperative complications" OR "population-based") |

**Table S2** Outcome definitions for individual studies.

| **Author, year** | **All-cause 30-day mortality** | **Intraoperative hypotension** | **Postoperative renal complications** |
| --- | --- | --- | --- |
| Chui, 2025 | NC | NA | The renal complications include the following ICD-10-CA diagnosis but not limited to: |
|  |  |  | - Glomerular disease |
|  |  |  | - Tubulo-interstitial disease |
|  |  |  | - Renal failure |
| Jerath, 2024 | Death within 30 days after surgery | NA | NA |
| von Wedel, 2024 | Death within 30 days of the index procedure | Mean invasively or non-invasively measured arterial blood pressure < 55 mmHg for a cumulative duration of ≥ 5-min | NA |
| Zeiner, 2024 | Death of all causes within 30 days | < 65 mmHg for at least 15-min | Acute Kidney Injury |

mmHg, Millimeters of mercury; Min, Minutes; NC, Not Collected (overlapping population); NA, Not Available.

**Table S3** Covariates included in adjusted models across included studies.

| **Author, year** | **Outcomes** | **Model type** | **Adjustment variables** |
| --- | --- | --- | --- |
| Chui, 2025 | Postoperative renal complications | Multivariable logistic regression | Patient sex; CCI; duration of surgery; emergency surgery; spinal cord tumor surgery; kyphosis/scoliosis correction; instrumentation/fusion; surgeon annual spine volume; surgeon subspecialty (neurosurgery vs orthopedic). (Age not included separately; incorporated in CCI) |
| Jerath, 2024 | 30-day mortality | Generalized estimating equations with multivariable regression | Patient-level, surgeon-level, anesthesiologist-level, and hospital-level covariates and year of surgery. |
| von Wedel, 2024 | 30-day mortality; Intraoperative hypotension | Generalized estimated equations with logistic regression models | Patient-level: age; patient sex; ASA physical status; Elixhauser comorbidity index; BMI; estimated household income; federal insurance status; admission type; proximity to hospital (≤ 10 miles).  Procedure/intraop-level: procedural service; emergency status; anesthesia care type (GA/MAC/RA); scheduled duration of surgery; work relative value units (surgical complexity).  Time/center: year of surgery; study center.  Provider-level: sex of primary surgical provider; anesthesia provider experience (prior case volume before index case); provider training level (trainee vs. non-trainee). |
| Zeiner, 2024 | 30-day mortality; Intraoperative hypotension; Postoperative renal complications | Linear mixed model | Patient sex; age; BMI; ASA score; duration of anesthesia; duration of anesthesia in concordance with ASA score; surgeon sex; CCI; number of previous general anesthesia cases by the provider. |

CCI, Charlson Comorbidity Index; BMI, Body Mass Index; ASA, American Society of Anesthesiologists; GA, General Anesthesia; MAC, Monitored Anesthesia Care; RA, Regional Anesthesia.

**Figure** **S1** Risk of bias assessment using the ROBINS-I tool for non-randomized studies.


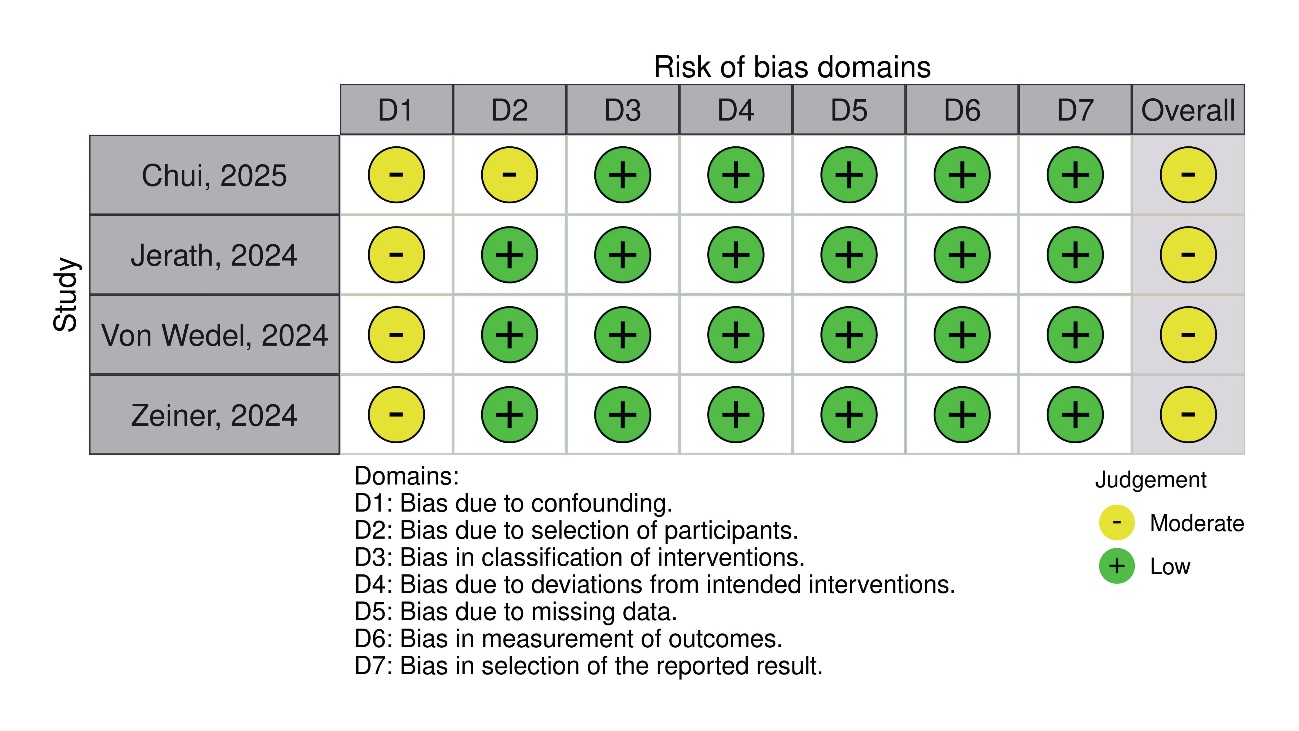


**Figure S2** PRISMA flow diagram of study screening and selection.

**
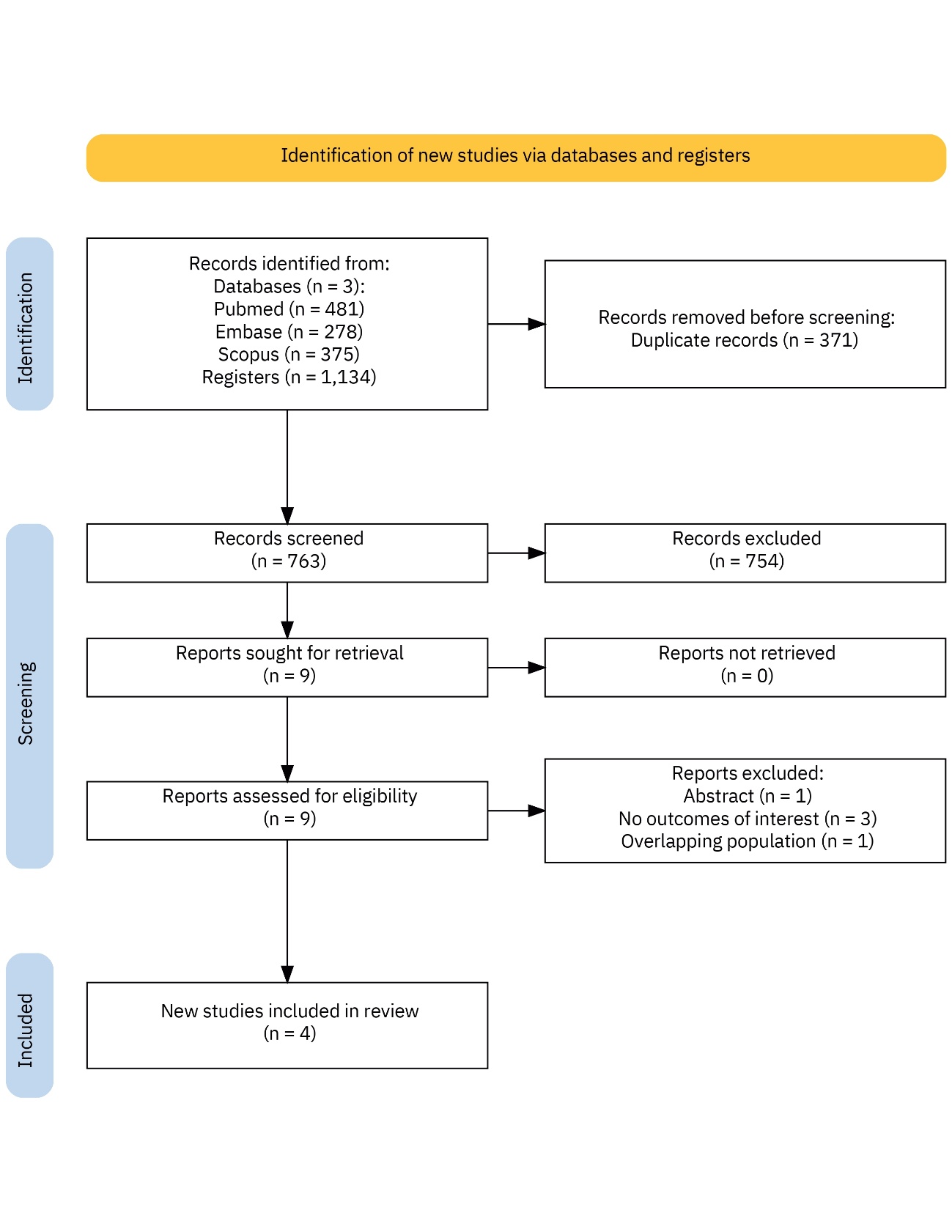
**
